# Fucosyltransferase 4 Predicts Patient Outcome in Rectal Cancer through an Immune Microenvironment-Mediated Multi-Mechanism

**DOI:** 10.1155/2022/4637570

**Published:** 2022-09-17

**Authors:** Chengqian Lv, Kunpeng Luo, Shuqiang Liu

**Affiliations:** ^1^Department of Gastroenterology and Hepatology, Second Affiliated Hospital of Harbin Medical University, Harbin, China; ^2^Department of Hepatopancreatobiliary Surgery, Second Affiliated Hospital of Harbin Medical University, Harbin, China

## Abstract

Colorectal cancer is the most common type of gastrointestinal malignant tumors worldwide. Standardization of the strategy for the precise treatment of this cancer has been a major challenge. Enrichment analysis of six gene groups (colon cancer-specific genes (upregulated and downregulated); rectal cancer-specific genes (upregulated and downregulated); and common genes (upregulated and downregulated)) revealed the common and specific features of colon and rectal cancer, particularly a hyperactive immune response in rectal cancer. Key common genes exhibited a similar expression pattern, but were associated with distinct patient prognosis in colon and rectal cancer. FUT4 was a core regulatory gene in rectal cancer; it can decrease the level of infiltration by M2 macrophages in the tumor immune microenvironment and participate in the positive regulation of the immune system and glycoprotein biosynthetic process, thereby affecting the outcome of patients with rectal cancer. FUT4 co-expression genes can influence patient's survival time by regulating the cell cycle. Among the regulators of FUT4 co-expression genes, checkpoint kinase 2 (CHEK2) was linked to patient outcome.

## 1. Introduction

Colorectal cancer (CRC) is the most common type of gastrointestinal cancers [1]. The incidence of CRC has sharply increased due to the shifting of disease risk factors over the past decade, especially among individuals aged <50 years [2, 3]. Despite advances in endoscopy, surgery, chemotherapy, and immunotherapy, current therapeutic modalities are unable to adequately meet the requirements of clinical treatment [4]. Recent research studies demonstrated that heterogeneity in molecular features and alterations in gene expression are the core contributors to the high incidence rate of CRC observed in adolescents [3]. Thus, there is an urgent need to further understand the biological characteristics of CRC cells.

Colon and rectal cancers exhibit different biological characteristics. Therefore, it is suggested that these two types of cancer should be treated differently [5]. The precise treatment of CRC demands a more detailed interpretation of the different characteristics of colon and rectal cancers. Furthermore, it has been proposed to abandon the term “colorectal cancer” due to differences in the anatomy, epidemiology, molecular carcinogenesis, and clinical features of colon and rectal cancers, as well as focus research efforts on their differential treatment [6]. This highlights the current major obstacle to the precise treatment of CRC. Differences in anatomy, embryonic origin, and intestinal flora characteristics can be useful in interpreting the differences between colon and rectal cancers; however, these factors may not be sufficient. Thus, revealing the shared and different features between colon and rectal cancers and identifying the critical genes and pathways involved in these conditions may help explore the potential diagnostic and therapeutic target for disease.

High-throughput RNA sequencing provides the opportunity to detect alterations in the gene expression pattern between the tumor and normal tissue. Analysis of RNA sequencing data can assist in identifying the different mechanisms underlying RNA expression across different types of tumors. In recent years, several studies have investigated differences in the microsatellite instability status, gene expression status, gene methylation status, and therapeutic responsiveness between CRCs of varied anatomical origin [7–9]. However, there is lack of integrated analysis of the gene expression pattern and its related biological functions in colon and rectal cancers. Thus, we conducted an integrated analysis to fill this gap in research.

## 2. Materials and Methods

### 2.1. Differentially Expressed Gene (DEGs) Acquisition and Related Analysis

The GEPIA2 database (http://gepia2.cancer-pku.cn/) is an integrated online-analysis tool for the expression of differentially expressed gene (DEGs) in cancer based on The Cancer Genome Atlas and Genotype-Tissue Expression database. This tool provides customizable analysis, including differential expression analysis, profiling plotting, correlation analysis, and patient survival analysis [10]. Samples with gene expression level at top 70% were considered as high expression group, and the other 30% samples were considered as low expression group. In this study, we utilized GEPIA2 to identify DEGs in the two types of gastrointestinal cancers, determine their chromosomal location, and conduct comparisons of the expression of multiple genes and their relationship with survival.

### 2.2. Functional Analysis and Visualization of Results

Gene Ontology (GO) and Kyoto Encyclopedia of Genes and Genomes (KEGG) enrichment analyses were performed to analyze the function of each gene group. We used clusterProfiler, the package of R software, to identify the molecular function, biological process, and cellular component for the GO analysis and pathway for the KEGG analysis [11]. The result was visualized in a bubble chart using the R package ggplot.

### 2.3. Construction of a Protein–Protein Interaction Network and Module Analysis

The protein–protein interaction network was constructed using the Search Tool for the Retrieval of Interacting Genes (STRING; http://string-db.org) (version 11.0) online database [12]. Interactions with a combined score > 0.4 and false discovery rate < 0.05 were included. Cytoscape (version 3.8.0) is an open source bioinformatics software with multi-plug-in apps for analyzing molecular interaction networks [13]. The hub genes of the network were identified using CytoHubba (version 0.1), a plugin-in application of the Cytoscape (version 3.8.0) software [14]. Here, we identify the genes interaction network's core module by MCODE in the parameters: degree cut − off = 2, node score cut − off = 0.2, k − score = 2, and *Max* depth = 100; then, the module with the most MCODE scores is considered the core module. The nodes are ranked by Maximal Clique Centrality (MCC) value. To further analyze the module and function of the hub genes, we conducted enrichment analysis using the Cytoscape plug-in application ClueGO (version 2.5.7) and CluePedia (version 1.5.7) [15, 16]. Only enriched terms with a *P* value < 0.05 were selected.

### 2.4. UALCAN Database Analysis

The UALCAN (http://ualcan.path.uab.edu) database is an integrated online-analysis tool for cancer omics data; this tool can be used to perform an expression analysis stratified according to characteristics [17]. Using UALCAN, in this study, we conducted an expression analysis stratified according to the clinical features and tumor protein p53 (TP53) mutation status.

### 2.5. Immune Infiltration Related Analysis

TIMER2.0 (http://timer.cistrome.org/) is a comprehensive resource for the systematic analysis of immune infiltration in cancer [18–20]. It provides information regarding the abundance of immune infiltrates, which is estimated by multiple immune deconvolution methods including TIMER [18], XCell [21], MCP-counter [22], CIBERSORT [23], EPIC [24], and QUANTISEQ [25]. In addition, it allows the generation of figures for the comprehensive exploration of immunological, clinical, and genomic features of tumors. Using TIMER2.0, in this study, we analyzed the fucosyltransferase 4 (FUT4)-mediated immune microenvironment and its relationship with the overall survival of patients. During the analysis of the overall survival of patients, parameters of TIMER2.0 were set as follow: split infiltration percentage of patients: (%) 30 and survival time between: 120.

### 2.6. Analysis of FUT4's Potential Coregulators and Their Biological Function

The LinkedOmics database (http://www.linkedomics. org/login.php) is a platform based on The Cancer Genome Atlas for the analysis and comparison of cancer multi-omics data within and across multiple types of tumors [26]. In this study, LinkedOmics was used to identify the FUT4 co-expression genes and analyze their function, as well as determine regulators of these co-expression genes. Co-expression genes' identification was conducted by Pearson correlation analysis. A *P* value < 0.05 and false discovery rate < 0.05 were set as thresholds for the identification of co-expression genes. This database was also used for the detection of GO-biological process, GO-cellular component, GO-molecular function, and KEGG pathways, as well as kinase-target identification, miRNA-target identification, and transcription factors– (TFs–) target identification through gene set enrichment analysis (GSEA) [27].

### 2.7. External Validation Set Related Analysis

To validate FUT4 and CHEK2's impact on patient's outcome, external validation set GSE87211 was enrolled in this research. The data series' clinic information and transcription data were acquired from Gene Expression Omnibus data base (GEO). Patients were divided into high expression group/low expression group according to FUT4 or CHEK2's expression level based on the best-separation cutoff value. Since patient's 5-year survival probability is commonly applied to assess patient's outcome in clinic practice, only the patient's 5-year follow-up information was enrolled in the research. Patient's outcome was compared by the R package “ggsurvplot.”

### 2.8. Cell Culture and Quantitative Real-Time PCR Assay

Human rectal cancer cell line SW480 and human primary rectal epithelial cell line were purchased from Saibaikang Biotechnology, Shanghai, China. Human primary rectal epithelial cell line was cultured in a humidified incubator with 5% CO2 at 37 °C in ICell Primary Epithelial Cell Culture System (Saibaikang Biotechnology, Shanghai, China). Human rectal cancer cell line SW480 was cultured in a humidified incubator with 5% CO2 at 37 °C in special culture medium for SW480 cells (Saibaikang Biotechnology, Shanghai, China). qRT-PCR analysis was carried out according to the published literature [28]. First-Strand cDNA Synthesis Kit and 2× SYBR Green qPCR Master Mix were purchased from SEVEN Biotechnology, Beijing, China. GAPDH was used as the internal control. Relative mRNA levels were calculated using the −*ΔΔ*Ct method and presented as 2(−*ΔΔ*Ct). Primers were purchased from Tongyong Biotechnology, Anhui, China. The primers were as follow: FUT4: forward: 5′-GATCTGCGCGTGTTGGACTA-3′;

reverse: 5′-GAGGGCGACTCGAAGTTCAT-3′;

GAPDH: forward: 5′-GGAGCGAGATCCCTCCAAAAT-3′;

reverse: 5′-GGCTGTTGTCATACTTCTCATGG-3′.

### 2.9. Statistical Analysis

A |logFC| > 2 and *P* value < 0.05 were set as thresholds for the identification of DEGs. The LIMMA package was applied for differential analysis. The correlation between FUT4 and co-expression genes was assessed using the Pearson correlation coefficient. For the cell-line-based assay, *t* test was employed to compare the two groups. If not specially mentioned, the comparison of survival curves, hazard ratios, and log-rank *P* values were calculated using the log-rank test.

## 3. Results

### 3.1. Schematic Diagram of the Overall Design

**Figure 1:**
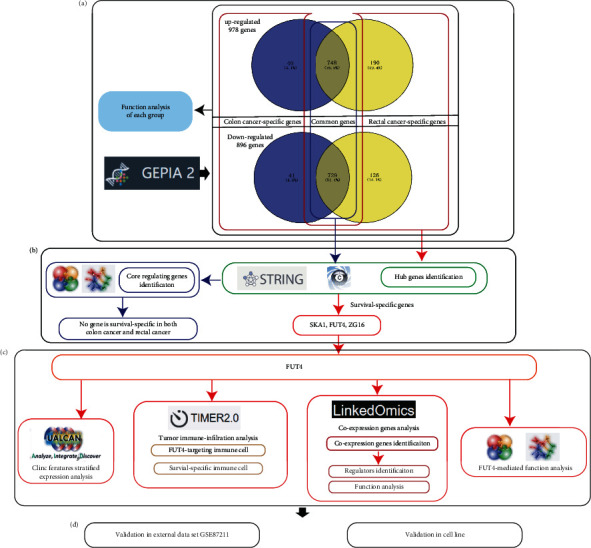
Flowchart of this study. (a) Analysis of shared and distinct features between colon and rectal cancers. (b) Identification of core regulatory genes. (c) Analysis of the regulatory role of FUT4 from multiple perspectives. FUT4: fucosyltransferase 4.

First, we determined DEGs between the colon and rectal cancers and normal tissue. The DEGs were divided into six groups, and a functional analysis of each group was subsequently conducted to analysis the biological heterogeneity and common features in the colon cancer and rectal cancer (Figure 1(a)). Next, we identified hub genes for each group and analyzed their impact on survival to detect the potential key regulatory genes (Figure 1(b)). Then multiplatforms were applied to analyze the rectal cancer-specific core regulatory gene FUT4's mediated immune microenvironment, network of co-expression genes, clinic feature's stratified expression status, and mediated function in order to roundly describe the role of FUT4 in rectal cancer (Figure 1(c)). Finally, external validation set and cell-line assay were conducted to validate FUT4's therapeutic potential (Figure 1(d)).

### 3.2. DEG Data Acquisition and Grouping

**Figure 2:**
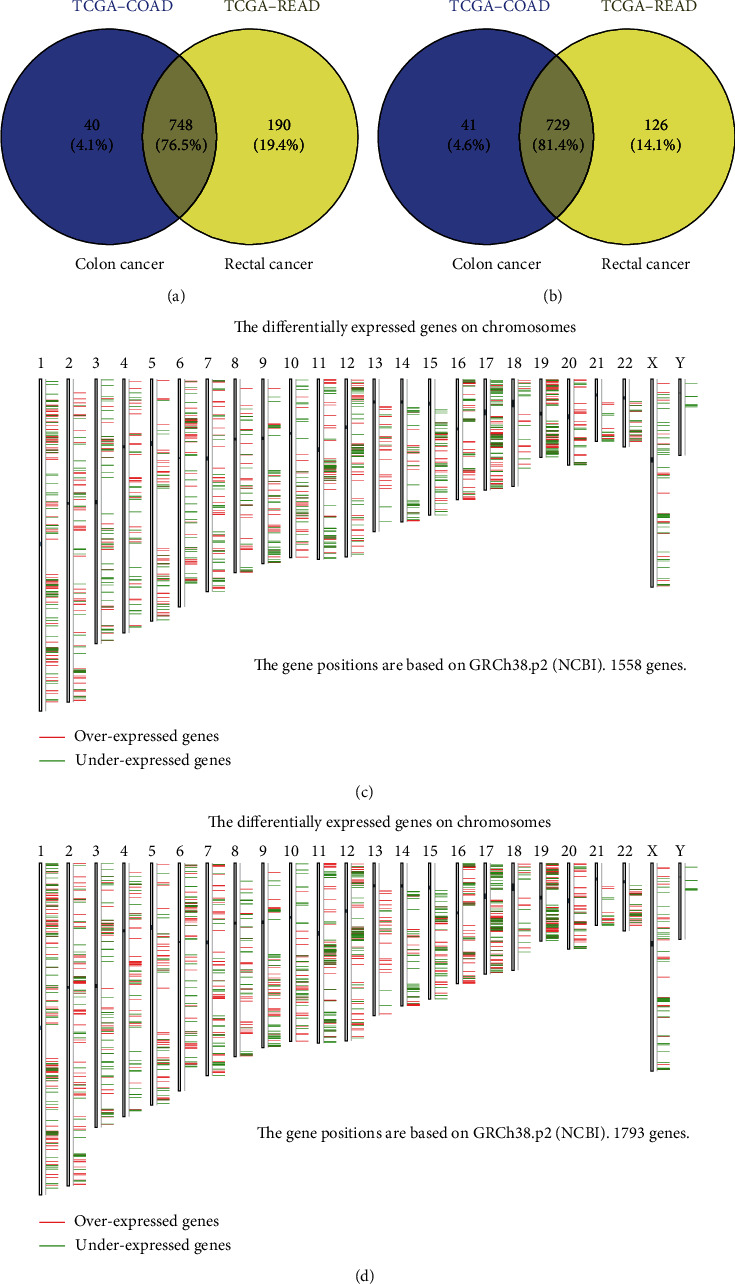
Identification of DEGs between CRC and normal tissues. (a) Upregulated and (b) downregulated DEGs in colon and rectal cancers based on data obtained from the GEPIA2. The overlapping areas represent common genes altered in both types of cancer. Chromosomal distribution of DEGs in (c) colon cancer and (d) rectal cancer. CRC: colorectal cancer; DEG: differentially expressed gene; GEPIA2: Gene Expression Profiling Interactive Analysis 2.

First, we collected the DEG data from GEPIA2. Subsequently, we divided the data into the following six groups: colon cancer-specific genes (upregulated and downregulated), rectal cancer-specific genes (upregulated and downregulated), and common genes (upregulated and downregulated) (Figures 2(a) and 2(b)). The chromosomal location of DEGs in the two diseases was also identified by GEPIA2 (Figures 2(c) and 2(d)). The results demonstrated that DEGs are similarly localized in these two types of cancer.

### 3.3. Function Enrichment of Each Gene Group

**Figure 3:**
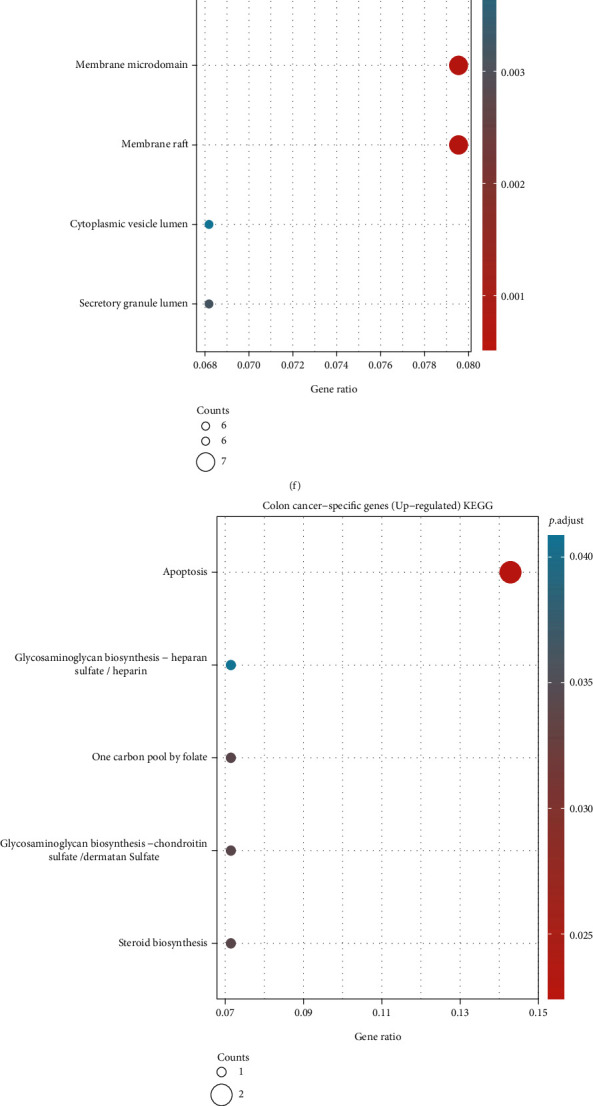
DEG-mediated function analysis. (a–f) Functional enrichment analysis of each gene group by GO. (g–l) Functional enrichment analysis of each gene group by KEGG. DEG: differentially expressed gene; GO: gene ontology; KEGG: Kyoto Encyclopedia of Genes and Genomes.

The GO analysis showed that genes in the colon cancer-specific genes (upregulated) group were mainly associated with the generation of epithelial features of cancer cells (Figure 3(a)). This result suggests that colon cancer is more epithelial-like and would be more sensitive to specific therapeutic strategies targeting cancers of epithelial origin compared with rectal cancer. The GO and KEGG analyses showed that genes in the common genes (upregulated) were mainly associated with the proliferation of cancer cells, particularly during the process of karyokinesis (Figures 3(b) and 3(h)). The result revealed the common overactivated karyokinesis pattern and high proliferative activity of the two types of cancer. Furthermore, the analyses showed that genes in the rectal cancer-specific genes (upregulated) group were mainly associated with hyperactive immune response (Figures 3(c) and 3(i)). Additionally, the enriched terms focused on antibody- and complement-mediated immune response, which is an immune response synergistically regulated by multiple types of immune cells. Thus, in the following analysis, we attempted to interpret the distinct immune pattern of rectal cancer by identifying the immune cells participating in this process. GO and KEGG analyses showed that genes in the colon cancer-specific genes (downregulated) group were mainly associated with cancer cell adhesion (Figures 3(d) and 3(j)). This result suggests that colon cancer is characterized by looser cell adhesion than rectal cancer. Additionally, the analyses showed that genes in the common genes (downregulated) group were mainly associated with contraction of vascular smooth muscle (Figures 3(e) and 3(k)). According to the results, the two gastrointestinal cancers exhibit similar histological heterogeneity compared with normal tissue. The GO and KEGG analyses showed that genes in the rectal cancer-specific genes (downregulated) group were mainly associated with the microstructure of the cell membrane and the circadian rhythm of cells (Figures 3(f) and 3(l)). The results presented the loss of the normal microstructure and circadian rhythm during the malignant transformation and dedifferentiation of rectal cells.

### 3.4. Common Hub Genes with Similar Expression Patterns Are Associated with Distinct Outcomes in Patients

Because colon and rectal cancers exhibit similar gene expression patterns (Figure 2), we further investigated their common characteristics. For this purpose, we selected the two common gene groups for further investigation of biological patterns.

**Figure 4:**
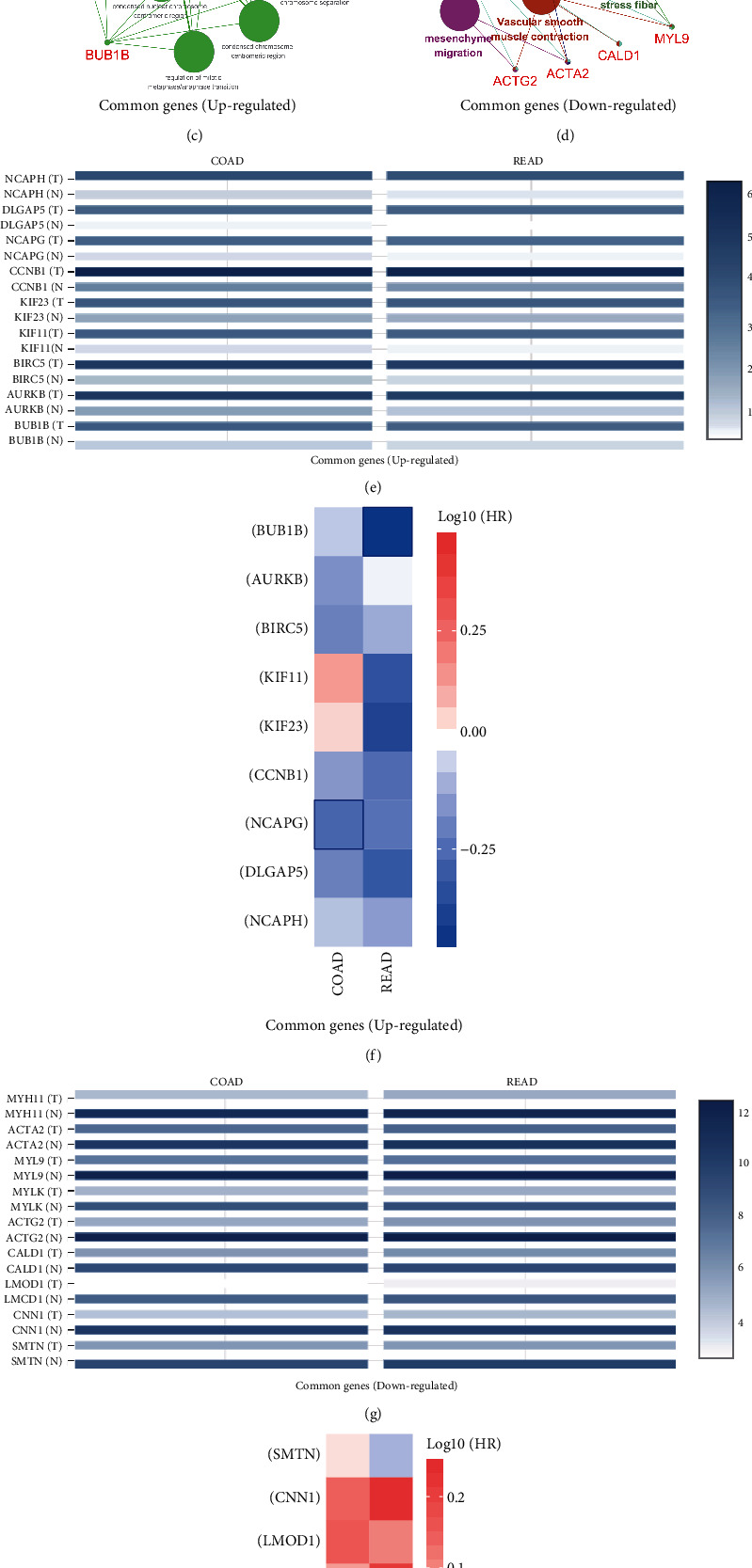
Integrated analysis of hub genes. Hub genes in the (a) upregulated and (b) downregulated groups. Function enrichment analysis of hub genes in the (c) upregulated and (d) downregulated groups. The expression level of key genes in the (e) upregulated and (g) downregulated groups in tumor tissues and normal tissues. Impact of hub genes on survival in the (f) upregulated and (h) downregulated groups. The blue boxes indicate a significant impact on patient survival (*P* < 0.05).

Firstly, we constructed the protein–protein interaction network using STRING and identified the top 10 hub genes of the two groups (Figures 4(a) and 4(b)). Subsequently, we conducted enrichment analysis of the hub genes to achieve a further interpretation of the biological function shared by these cancers (Figures 4(c) and 4(d)). Most hub genes of the common genes (upregulated) group were enriched in the mitotic sister chromatid segregation function, revealing the common hyperactive proliferation pattern of the two types of cancer (Figure 4(c)). This finding is consistent with the results of our enrichment analysis of genes in the common genes (upregulated) group (Figures 3(b) and 3(h)). Most hub genes of the common genes (downregulated) group were enriched in the vascular smooth muscle contraction function, revealing common histological heterogeneity of the two cancers versus normal tissue (Figure 4(d)). This result is also consistent with those of the enrichment analysis of genes in the common genes (downregulated) group (Figures 3(e) and 3(k)). Among the hub genes identified, 18 genes were enriched to the particular terms which referred to their essential regulatory roles in the disease. Therefore, we further analyzed the expression pattern of these 18 genes (Figures 4(e) and 4(g)) and their impact on survival (Figures 4(f) and 4(h)). Interestingly, these genes exhibited similar expression patterns in the two types of tumors but had limited impact on the survival of patients with these cancers. To explain this observation, we focused on the remaining four groups. Thus, we hypothesized that the nonoverlapping DEGs, which constitute a smaller proportion than the common DEGs, result in differences in the survival patterns linked to these cancers.

### 3.5. FUT4 Is the Core Regulatory Gene That Predicts Outcome in Patients with Rectal Cancer

**Figure 5:**
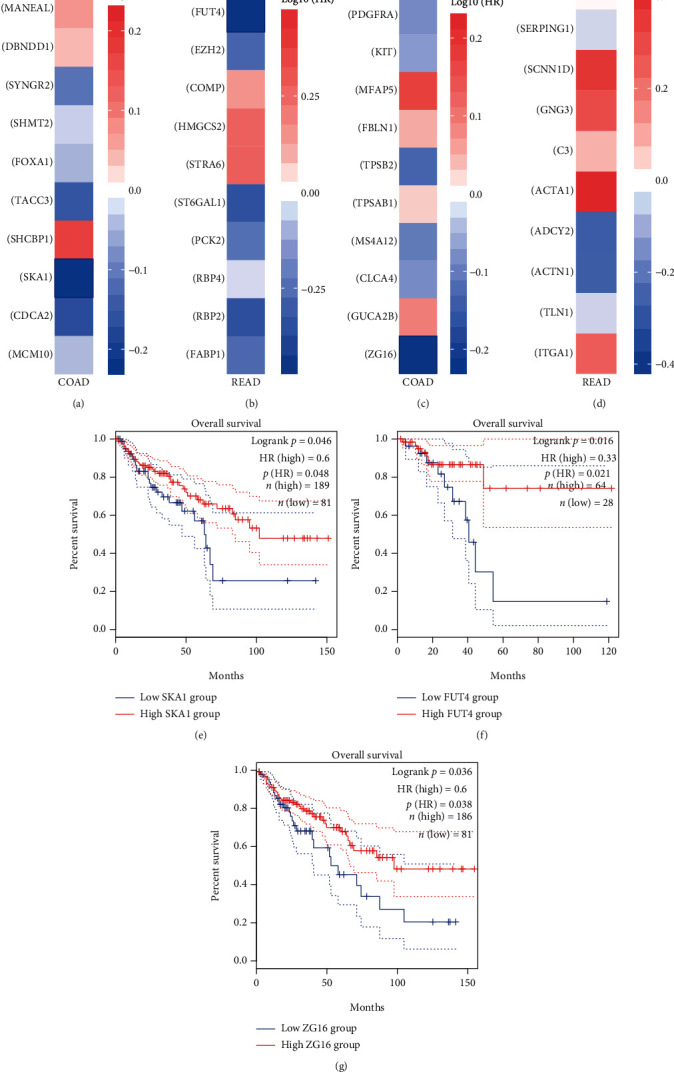
Identification of key regulatory genes. HR of the top 10 hub genes in the (a) colon cancer-specific genes (upregulated) group, (b) rectal cancer-specific genes (upregulated) group, (c) colon cancer-specific genes (downregulated) group, and (d) rectal cancer-specific genes (downregulated) group. The blue boxes indicate a significant impact on survival. Kaplan–Meier curves depicting the survival impact of (e) SKA1, (f) FUT4, and (g) ZG16. The blue boxes indicate a significant impact on patient survival (*P* < 0.05). FUT4: fucosyltransferase 4; HR: hazard ratios; OS: overall survival; SKA1: spindle and kinetochore associated complex subunit 1; ZG16: zymogen granule protein 16.

**Figure 6:**
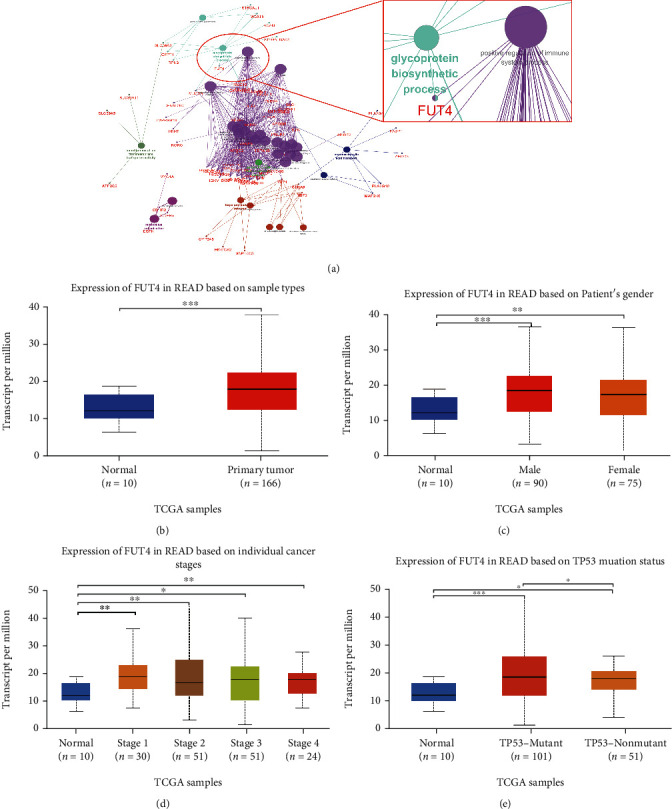
FUT4 function expression analysis stratified by clinical features. (a) Function analysis of the regulatory role of FUT4. (b–e) The expression of FUT4 stratified by different sample types, sex, individual cancer stage, and TP53 mutation status. ^∗^*P* < 0.05; ^∗∗^*P* < 0.01; and ^∗∗∗^*P* < 0.001. FUT4: fucosyltransferase 4; TP53: tumor protein p53.

To validate our hypothesis, we conducted further analysis of the remaining four genes groups. Initially, we generated the protein–protein interaction network using STRING and identified the hub genes of each group by CytoHubba. Subsequently, we tested the impact of each gene on survival using data from GEPIA2 (Figures 5(a)–5(d)). Among all the hub genes, only spindle and kinetochore associated complex subunit 1 (SKA1), FUT4, and zymogen granule protein 16 (ZG16) were correlated with patient survival. Among these three genes, FUT4 demonstrated the greatest and most significant impact on patient outcome, according to the hazard ratio and *P* value (Figures 5(e)–5(g)). Thus, we selected FUT4 as the core regulatory gene for further investigation. To reveal the distinct role of FUT4 in rectal cancer, we analyzed FUT4-mediated function in the rectal cancer-specific genes (upregulated) group. According to the findings, FUT4 participated in the “positive regulation of immune system process” and “glycoprotein biosynthetic process” in rectal cancer (Figure 6(a)). Recent studies showed that members of the glycoprotein family regulate several antitumor processes in CRC through immune cell-mediated and immunoglobulin-mediated mechanisms [29–31]. Thus, based on the results of the enrichment analysis, we hypothesized that FUT4 plays a regulatory role in outcome in patients with rectal cancer by targeting immune-related processes and contributing to the tumor immune microenvironment.

We also analyzed the correlation between FUT4 expression and clinical features using the UALCAN database. Significant differences in the expression of FUT4 were found between patients with rectal cancer and normal controls, as well as in sex, cancer stage, and TP53 mutation subgroups (Figures 6(b)–6(e)).

### 3.6. FUT4 Expression Predicts Patient Outcome through the M2 Macrophage-Mediated Mechanism in Rectal Cancer

**Figure 7:**
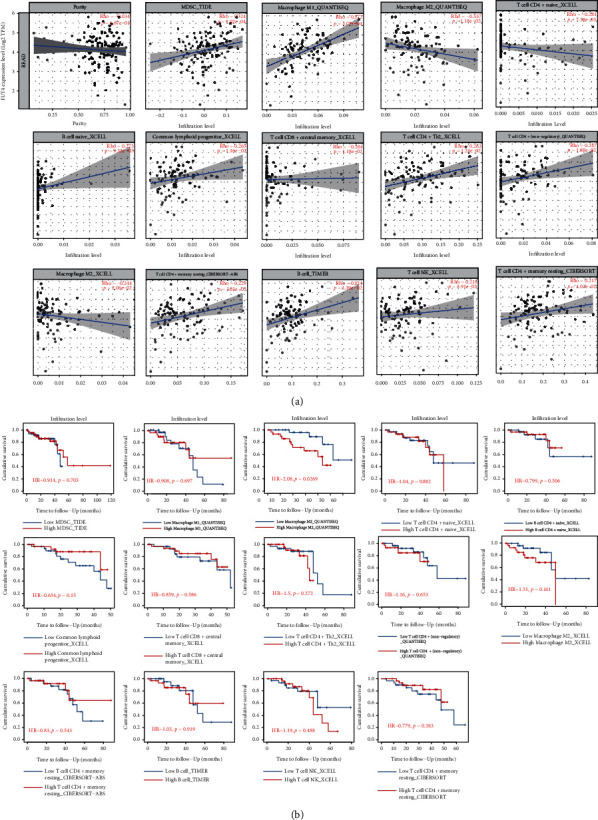
Analysis of the FUT4-mediated immune microenvironment. (a) Correlation between the expression level of FUT4 and infiltration level of different immune cells in the immune microenvironment. (b) Cumulative survival analysis in patients with different immune-cell concentration statuses. FUT4: fucosyltransferase 4.

The tumor-infiltrating immune cells are important markers of patient outcome in cancer. Hence, we assessed the correlation between the levels of immune infiltration and FUT4. This assessment was performed using TIMER2.0 to validate the hypothesis that FUT4 regulates biological function and consequently predicts patient outcome by influencing the infiltration levels of certain types of immune cells. Our results showed that the expression of FUT4 was significantly positively correlated with the infiltration of myeloid-derived suppressor cells (TIDE), macrophage M1 (QUANTISEQ), B cell naïve (XCELL), common lymphoid progenitor (XCELL), T cell CD4+ T helper 2 (XCELL), T cell CD4+ (non-regulatory) (QUANTISEQ), T cell CD4+ memory resting (CIBERSORT-ABS), B cell (TIMER), T cell natural killer (XCELL), and T cell CD4+ memory resting (CIBERSORT) cells. Moreover, it was negatively correlated with the infiltration of macrophage M2 (QUANTISEQ, XCELL), T cell CD4+ naïve (T cell CD4+ naive), and T cell CD8+ central memory (XCELL) cells (Figure 7(a)). The recorded immune-infiltration pattern was consistent with the results of the enrichment analysis for the rectal cancer-specific genes (upregulated) group (Figures 3(c) and 3(i)). The most enriched term in the GO “immunoglobulin complex” was the B cell-mediated bioprocess. This result was consistent with the positive correlation of FUT4 with the level of B cell infiltration. During the process of immunoglobulin complex generation, macrophages M1 and M2 play upregulating and downregulating roles, respectively; these findings were also consistent with the correlations of FUT4 [32, 33]. The most enriched term in GO (i.e., “Fc gamma R-mediated phagocytosis”) is also a B cell-produced immunoglobulin and macrophage-mediated immune process. This result was also consistent with the correlation of immune infiltration and FUT4 [32–34]. The other enriched immune-related terms were also in agreement with the relative immune-infiltration pattern of FUT4. The above findings confirm our hypothesis that FUT4 exerts a regulatory effect and affects survival by regulating the immune process and immune cell infiltration in the tumor immune microenvironment.

To further validate the impact on outcome, we analyzed the FUT4-mediated infiltration of all immune cells related to patient survival. The patients were divided into two groups according to their level of immune infiltration. Only macrophages M2 (QUANTISEQ) were associated with significant differences in outcome between the high- and low-infiltration groups (Figure 7(b)) Moreover, the correlation between FUT4 and macrophages M2 was the only one to be validated in both databases (QUANTISEQ, XCELL). The results indicated that FUT4 is a regulator of macrophage M2 infiltration to predict patient outcome. According to TIMER2.0 and enrichment analyses of the rectal cancer-specific genes (upregulated) group, other immune cells such as myeloid-derived suppressor cells, macrophage M1, B cell naïve, common lymphoid progenitor, T cell CD4+ T helper 2, T cell CD4+ (non-regulatory), T cell CD4+ memory resting, B cell, T cell natural killer, T cell CD4+ memory resting, T cell CD4+ naïve, and T cell CD8+ central memory cells play important regulatory roles in the tumor immune microenvironment (Figures 3(c), 3(i), and 6(a)).

### 3.7. Identification of FUT4 Co-Expression Genes

**Figure 8:**
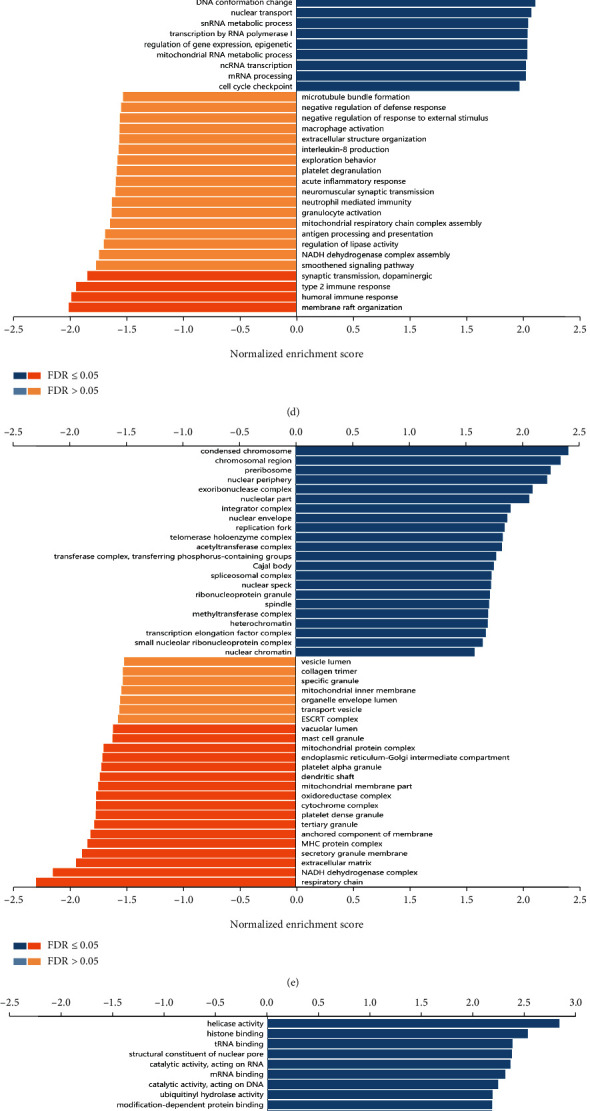
Analysis of FUT4 co-expression genes. (a) Heat map of the top 50 FUT4 co-expression genes. (b) Functional enrichment analysis of the top 50 FUT4 co-expression genes. (c) HR values of the top 50 FUT4 co-expression genes in colon and rectal cancers. The blue boxes indicate a significant impact on patient survival. GSEA enrichment analysis of FUT4 co-expression genes using the (d) GO-BP, (e) GO-CC, (f) GO-MF, and (g) KEGG modules. FUT4: fucosyltransferase 4; GO: gene ontology; GO-BP: GO-biological process; GO-CC: GO-cellular component; GO-MF: GO-molecular function; GSEA: gene set enrichment analysis; HR: hazard ratio; KEGG: Kyoto Encyclopedia of Genes and Genomes.

To further investigate the potential role of FUT4 in colon and rectal cancers, we detected its co-expression genes and performed functional enrichment analysis. The top 50 significantly correlated genes are presented in Figure 8(a). The function of these genes was determined using ClueGO. According to the results of the analysis, FUT4 co-expression genes mainly participate in cancer cell proliferation and gene expression (Figure 8(b)). To identify the impact of co-expression genes on the outcome of patients with colon and rectal cancers, we plotted the survival map of the top 50 significant correlated genes identified in GEPIA2 (Figure 8(c)). For genes which were not identified by this analysis, we selected the next co-expression gene. Notably, it performed the significant likelihood that top 50 correlated genes are protective genes in patients with rectal cancer. Of those, twenty genes and one gene were significantly correlated with better outcome in rectal and colon cancer, respectively. This result may explain the distinct impact of common key regulatory genes in colon and rectal cancer on survival, despite their similar expression patterns.

We also conducted enrichment analysis of all co-expression genes through GSEA (Figures 8(d)–8(g)). The top three most enriched terms in GO-biological process modules were RNA localization, regulation of mRNA metabolic process, and ncRNA processing. The top three most enriched terms in GO-cellular component modules were condensed chromosome, chromosomal region, and preribosome. The top three enriched terms in GO-molecular function were helicase activity, histone binding and catalytic activity, and acting on RNA. The top three most enriched terms in KEGG were ribosome biogenesis in eukaryotes, RNA transport, and aminoacyl-tRNA biosynthesis. The integrated results suggested that the FUT4 co-expression genes perform their regulatory function by exerting a broad effect on nuclear activity and gene translation in tumor cells. This result is also consistent with the function that the top 50 FUT4 co-expression genes mediated (Figure 8(b)).

### 3.8. Regulators of FUT4 Co-Expression Genes in Rectal Cancer

**Figure 9:**
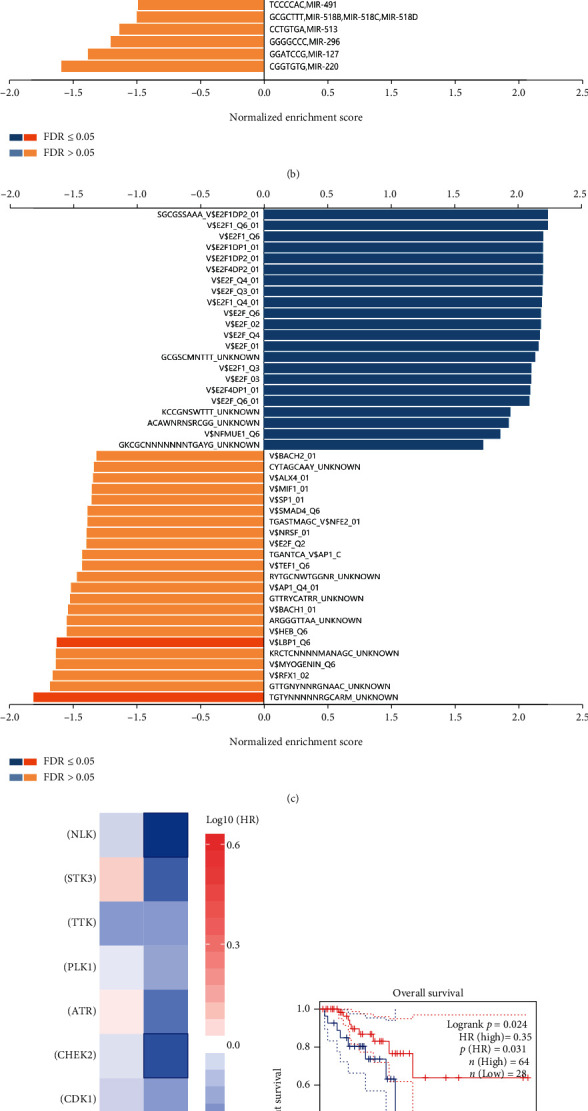
Analysis of regulators of FUT4 co-expression genes. The enrichment status of regulatory (a) miRNAs, (b) kinases, and (c) TFs. (d) HR values of the top 10 regulatory kinases of FUT4 co-expression genes in the two types of cancer. The blue boxes indicate a significant impact on survival. Survival analysis of (e) CHEK2 and (f) NLK in patients with rectal cancer. The expression analysis of (g) CHEK2 and (h) NLK. CHEK2: checkpoint kinase 2; FUT4: fucosyltransferase 4; HR: hazard ratio; NLK: nemo-like kinase; TF: transcription factor.

We subsequently identified the regulators of FUT4 co-expression genes, including miRNAs, kinases, and TFs (Figures 9(a)–9(c)).

Proteins belonging to the kinase family are emerging regulators of several cellular processes in CRC (e.g., proliferation, migration, angiogenesis, invasion, and metastasis) by contributing to the signal transduction of cells [35–37]. Next, we further analyzed the impact of regulatory kinases of the FUT4 co-expression genes on survival. Among the top 10 regulatory kinases, only CHEK2 and nemo-like kinase (NLK) had a significant impact on outcome in patients with rectal cancer (Figure 9(d)); of note, none of these kinases had a significant influence on the outcome of patients with colon cancer. However, only CHEK2 was associated with both significantly higher expression in tumor tissue and significant impact on the outcome of patients with rectal cancer (Figures 9(g) and 9(h)). This suggests that CHEK2 may be a core regulatory kinase in rectal cancer, exerting its biological effect by regulating the expression of FUT4 co-expression genes.

### 3.9. Validation of the Prognostic Value of FUT4 and CHEK2 in External Validation Set

**Figure 10:**
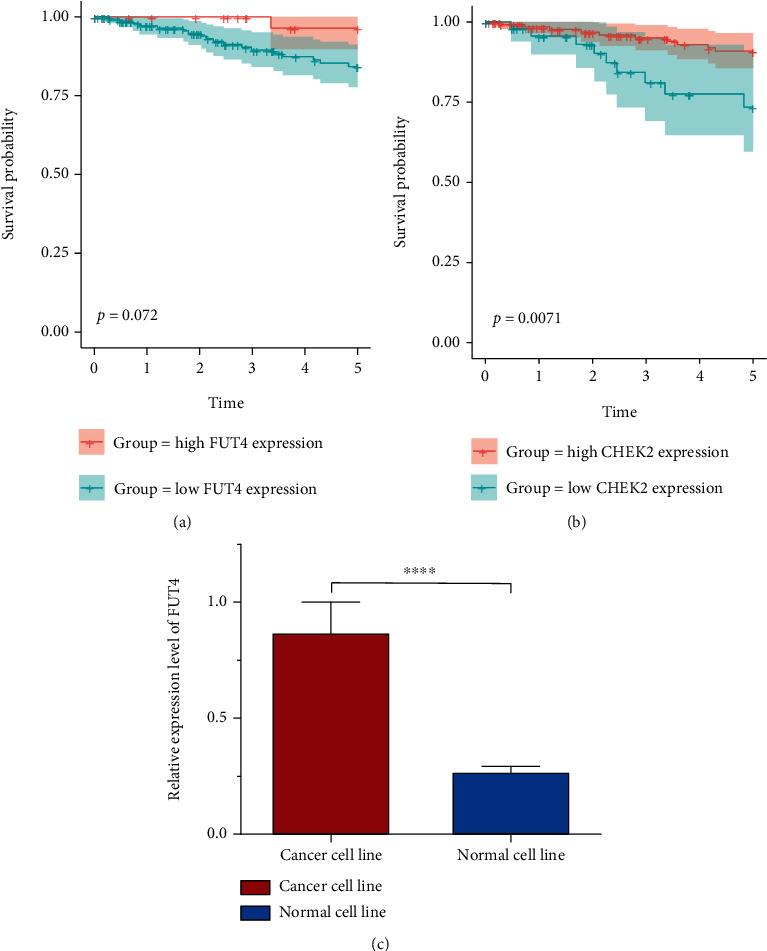
Validation of prognostic value of FUT4 and CHEK2 in GSE87211. Kaplan–Meier survival analysis of FUT4 (a). Kaplan–Meier survival analysis of CHEK2 (b). Comparison of FUT4's expression level in cancer cell line and normal cell line (c). ^∗∗∗∗^*P* < 0.0001.

To test the prognostic value of FUT4 and CHEK2, we compared the patient's outcome of high expression group and low expression group in GSE87211. Kaplan–Meier (K–M) survival curves demonstrated that FUT4 and CHEK2 can also relatively well predict patient's outcome in validation cohort (Figures 10(a) and 10(b)). FUT4's expression status was validated in cell line (Figure 10(c)). The results further proved the predicting accuracy of FUT4 and CHEK2.

## 4. Discussion

In recent years, radical changes have been observed in the dietary habits of young individuals. These changes have resulted in a distinct epidemiology and continuously rising incidence rate of CRC [38]. At present, there is a gap between the need for precise treatment of CRC and the current treatment strategy. Recent research studies focusing on the precise treatment of cancer and target identification proposed a new approach to cancer therapy and led to better patient outcomes [39–41].

The results of this study showed that most DEGs overlapped in the two types of cancer (Figures 2(a) and 2(b)) and exhibited similar chromosomal location patterns (Figures 2(c) and 2(d)). The GO and KEGG enrichment analyses illustrated a common hyperactive proliferation pattern and histology heterogeneity feature between the two gastrointestinal cancers (Figure 3). To further investigate common key regulators, we identified the top 10 hub genes among the common genes (upregulated and downregulated) (Figures 4(a) and 4(b)). From the hub gene network, 18 enriched genes which were identified as key regulatory genes (Figures 4(c) and 4(d)). However, none of those had a significant impact on patient outcome in both colon and rectal cancers (Figures 4(f) and 4(h)). There results imply that, although exclusive DEGs constitute a smaller proportion than common DEGs, they may play essential regulatory roles in the disease and predict different clinical outcome in patients with these two types of cancer (Figures 2(a) and 2(b)).

We subsequently sought to identify the key regulatory genes that may predict distinct patient outcomes in this setting. Among the 40 genes identified from the four exclusive DEGs group, only SKA1, FUT4, and ZG16 correlated with patient survival (Figures 5(e)–5(g)). Among those three genes, FUT4 was linked to the most significant *P* value and hazard ratio (Figure 5(f)). We also conducted an expression analysis for FUT4 stratified by clinical features (Figures 6(b)–6(e)), which revealed significantly elevated expression levels in rectal cancer. The significantly elevated expression of FUT4 in the TP53 mutation subgroups suggested that FUT4 may be a co-occurrence gene with this mutation (Figure 6(e)). The results of the enrichment analysis showed that FUT4 positively regulates the immune system process in patients with rectal cancer. Moreover, the significant differences in the expression of FUT4 between patients with a different TP53 mutation status imply that this mutation may lead to tumor occurrence and progression by contributing to the regulation of the tumor immune response. The results of a recent study also supported this hypothesis [42].

According to some studies, the function of FUT4 in CRC appears to be contradictory, i.e., linked to poor and good patient outcomes [43–45]. The complex role of FUT4 in CRC may be due to the investigation of both colon and rectal cancers in this study. The present results demonstrated the rectal cancer-specific regulatory role of FUT4 and revealed its specific regulatory mechanism in colon and rectal cancers. According to the results of the enrichment analysis of rectal cancer-specific genes, FUT4 exerts its regulating impact by upregulating the immune response (Figure 6(a)). Furthermore, GO and KEGG analyses of rectal cancer-specific genes also revealed a hyperactive immune response in rectal cancer, particularly the antibody- and complement-mediated immune response (Figures 3(c) and 3(i)). This finding suggests that FUT4 plays an important role in the tumor immune microenvironment. Based on the above results, the immune system is in a more hyperactive state in patients with rectal cancer versus patients with colon cancer, and FUT4 greatly contributes to this condition. Under this premise, we decided to analyze alterations in the immune microenvironment that may correlate with the expression levels of FUT4.

According to the analysis conducted using TIMER2.0, the expression of FUT4 is significantly correlated with infiltration of multiple types of immune cells in the tumor immune microenvironment (Figure 7(a)). Of note, FUT4 significantly correlated with both several antigen-presenting cells, including many types of macrophages and immunoglobulin-producing cells, such as B cells. This result demonstrated that FUT4 has great potential as a target for an mRNA vaccine against rectal cancer. The immunoglobulin- and complement-mediated immune response is the co-regulation bioprocess of the microenvironment for T cells, B cells, and antigen-presenting cells. Therefore, the correlations of FUT4 with the immune infiltration pattern and immune macroenvironment features are also consistent with the results of the enrichment analysis (Figures 3(c) and 3(i)). Among the immune cells that correlated with FUT4 expression, only M2 macrophages had a significant impact on patient outcome. Moreover, this correlation was the only one validated in two databases (xCell, quanTIseq) (Figure 7). Collectively, these results indicated that FUT4 decreases the level of infiltration of M2 macrophages to predict the outcome of patients with rectal cancer and induce a more active immune response in rectal cancer. This result is also consistent with the established immunosuppressive regulatory role of M2 macrophages in the CRC microenvironment [46–48].

To further investigate the regulatory mechanism of FUT4, we identified FUT4 co-expression genes and analyzed their influence on patient outcome. Among the top 50 co-expression genes, 21 genes and one gene had a significant impact on overall survival in patients with rectal and colon cancer, respectively (Figure 8(c)). This result further illustrates the regulatory role of FUT4 in rectal cancer. The GO and KEGG enrichment analyses for co-expression genes demonstrated that FUT4 can exert its regulatory power by influencing nuclear activity and gene translation in cancer cells (Figures 8(d)–8(g)). We also identified the regulators, including miRNA, kinase, and TFs using LinkedOmics (Figures 9(a)–9(c)). Among the top 10 regulatory kinases, only CHEK2 had a significant impact on overall survival and significantly elevated expression levels in tumor tissue (Figures 9(d)–9(h)). These results demonstrated that CHEK2 is the key regulatory kinase in the FUT4 co-expression gene network and predicts the outcome of patients with rectal cancer together with FUT4. This conclusion was also validated in the validation data set (Figures 10(a) and 10(b)). Meanwhile, FUT4's expression status was validated in cell line (Figure 10(c)).

**Figure 11:**
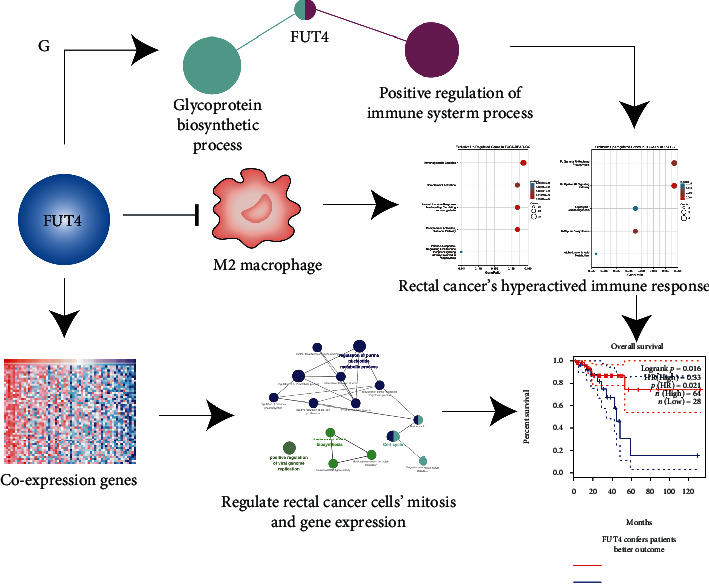
FUT4 predicted the outcome of patients with rectal cancer through an immune microenvironment-mediated multi-mechanism. FUT4 induced a hyperactive immune response in rectal cancer by decreasing the level of M2 macrophage infiltration and participated in the glycoprotein biosynthetic process and positive regulation of the immune system process. Moreover, FUT4 co-expression genes regulated the mitosis and gene expression in rectal cancer cells.

In rectal cancer, FUT4 predicts the outcome of patients through an immune macroenvironment-mediated mechanism. It downregulates the infiltration level of M2 macrophages and participates in the glycoprotein biosynthetic process and positive regulation of the immune system process to contribute to the hyperactive immune response in rectal cancer. Nevertheless, FUT4 co-expression genes regulate mitosis, gene translation, and gene transcription in cancer cells. Together, the two mechanisms confer better outcomes in patients with rectal cancer (Figure 11). The argument for abandoning the term “colorectal cancer” also appears to come to a conclusion [6]. The two types of cancer share similar patterns of differential gene expression gene, histological heterogeneity, and hyperactive proliferation. Moreover, most DEGs also overlap between the two cancers. Thus, the proposal to abandon the term “colorectal cancer” may be excessively radical. However, cancer-specific DEGs which constitute a smaller proportion also play critical regulatory roles in disease progression. Thus, analysis of their regulatory function and identification of potential therapeutic targets to meet the need for the precise treatment of CRC is warranted. The present study implies that colon and rectal cancers should be treated independently. The results of this study propose two potential genes as target candidates for the precise treatment of rectal cancer: the immune microenvironment regulatory gene FUT4 and kinase family member CHEK2.

## Data Availability

Publicly available data were analyzed in this study. The data presented in this research can be found in an online repository. The name of the repository is provided in the article.
